# Life in the hole: practices and emotions in the cultural political economy of mitigation deterrence

**DOI:** 10.1186/s40309-021-00186-z

**Published:** 2022-03-13

**Authors:** Nils Markusson, Duncan McLaren, Bronislaw Szerszynski, David Tyfield, Rebecca Willis

**Affiliations:** Lancaster University, Lancaster, UK

## Abstract

Negative emissions techniques (NETs) promise to capture greenhouse gases from the atmosphere and sequester them. Since decarbonisation efforts have been slow, and the climate crisis is intensifying, it is increasingly likely that removing greenhouse gases from the atmosphere will be necessary to meet internationally-agreed targets. Yet there are fears that pursuing NETs might undermine other mitigation efforts, primarily the reduction (rather than removal) of greenhouse gas emissions. This paper discusses the risk of this phenomenon, named ‘mitigation deterrence’. Some of us have previously argued that a cultural political economy framework is needed for analysing NETs. Such a framework explains how promises of future NETs deployment, understood as defensive spatio-temporal fixes, are depoliticised and help defend an existing neoliberal political regime, and its inadequate climate policy. Thus they risk deterring necessary emissions reductions. Here we build on that framework, arguing that to understand such risks, we need to understand them as the result of historically situated, evolving, lived practices. We identify key contributing practices, focussing in particular but not exclusively on climate modelling, and discuss how they have been reproduced and co-evolved, here likened to having dug a hole for ourselves as a society. We argue that understanding and reducing deterrence risks requires phronetic knowledge practices, involving not just disembodied, dispassionate technoeconomic knowledge-making, but also strategic attention to political and normative issues, as well as emotional labour. Reflecting on life in the hole hurts.

## Introduction

Given the urgent need to tackle anthropogenic climate change, much attention has been paid not only to reducing ongoing carbon dioxide emissions but also to techniques which promise to remove greenhouse gases from the atmosphere. Many companies and countries have now set targets for ‘net zero’ emissions. Under a net zero target, any carbon dioxide (and in some cases any greenhouse gas) emissions must be balanced by removals, so that there is no net increase in atmospheric levels of greenhouse gases. Thus ‘net zero’ targets set by countries or corporations incorporate negative emissions techniques (NETs^1^). Much modelling and forecasting work, such as the models used by the Intergovernmental Panel on Climate Change [23], also assumes the use of NETs in projections of how emissions can be eliminated in order to stabilise the climate.

NETs are techniques that promise to capture greenhouse gases, typically CO_2_, from the atmosphere and sequester them [42]. NETs are typically envisioned to be used as a complement to conventional emissions reduction (‘mitigation’). Examples of NETs include Biomass Energy with Carbon Capture and Storage (BECCS) and afforestation. No NET has yet been deployed at large scale for the purpose of removing greenhouse gases. Some techniques, like BECCS, need further development and demonstration, whereas others, like afforestation, are proven as such, but have not been deployed for carbon removal effectively at the vast scales implied by the idea of NETs.

There have long been fears that pursuing NETs might distract from the crucial task of reducing emissions at source. This has often been framed in terms of ’moral hazard’ [8, 10, 26]. We are however concerned that the ‘moral hazard’ term unduly narrows the scope to individual (techno-economically) rational decision making about well-characterised risks, and obscures impacts on mitigation that are collectively emergent, culturally mediated, politically driven, and/or partly unknown [31]. Thus, we use the broader term ‘mitigation deterrence’ (following [33]), defined as *the prospect of reduced or delayed emissions reduction resulting from the introduction or consideration of another climate intervention*.

We here set out to synthesise results from a project in which we sought to contribute an analysis of mitigation deterrence effects from NETs, as well as interventions to counter them, addressing the key question: ‘*under what conditions can NETs coexist with, and complement, other mitigation strategies?*’ Other papers forming part of this project have laid out a theoretical framework for considering mitigation deterrence, using the lens of cultural political economy (CPE) [31]; developed a typology of different forms of mitigation deterrence [36]; and taken a historical look at how, at each stage in the climate debate, different formulations of the problem have led to different forms of mitigation deterrence [35]. Other papers analyse reflections about mitigation deterrence risks from deliberative stakeholder workshops, and propose proposals for interventions [34, 37].

In this paper, we draw together the analysis from the project as a whole, and add a crucial dimension: a focus on the practices that evolve and are reproduced by stakeholders including policymakers, researchers, business interests, civil society voices and others. We liken the problem of mitigation deterrence to life in a hole—a hole which has been dug by all those involved. Armed with shovels, there is a strong temptation—indeed, an expectation—to keep digging. It seems like the best thing to do. To question the digging means questioning your own and others’ motivations, assumptions and ideas of success. We argue that understanding and reducing deterrence risks require phronetic knowledge practices; i.e. situated practical wisdom, involving not just disembodied, dispassionate techno-economic knowledge-making, which tends to depoliticise promises of future technology, but also strategic attention to political and normative issues, as well as emotional labour. Reflecting on life in the hole hurts.

The paper proceeds in three stages. First, we reinterpret the cultural political economy of mitigation deterrence framework in terms of practices, and we explore the notion of defensive spatio-temporal fixes (STFs) that link promises of future NETs deployment, (re)produced in climate modelling and other practices, to the wider political economy. Second, we explore in some detail how the framing of NETs in climate modelling practice enables mitigation deterrence, and discuss further how this and other contributing practices have been reproduced and co-evolved in a long-established deterrence dynamic. Third, we consider what would be involved in deliberately trying to reduce the risk of mitigation deterrence in the face of a recalcitrant political economy, including the emotional labour involved in reflecting on our commitments to that political economy.

## The cultural political economy of mitigation deterrence

This section sets out the conceptual starting points for the paper. We first summarise the origins and core features of the cultural political economy of mitigation deterrence [31], and explain this in terms of the notion of defensive spatio-temporal fixes that link promises of future NETs deployment, reproduced in climate modelling and other practices, to the wider political economy. We then turn to the crucial issue of practices.

### Cultural political economy and the lure of spatio-temporal fixes

As we have previously argued, NETs and associated mitigation deterrence need to be understood within the context of political regimes^2^ [30] and, specifically, through the lens of cultural political economy. Through that lens, we can analyse how technical fixes co-evolve with political regimes. In other words, certain technologies and approaches may be encouraged and promoted because they ‘fit’ within existing political and economic institutions, and others may be discouraged or side-lined because they do not align well.

A crucial aspect of the capitalist system is the way in which it responds to potential crisis situations. Harvey [19] defined the concept of ‘spatio-temporal fixes’ (STFs) to describe the ways in which capital defers its tendency to crisis, by shifting its problem to other places or times^3^. Specifically, when the capitalist system is faced with a surplus of accumulated capital, by defining and investing in new long-term projects capital can defer the moment when that surplus re-enters circulation; and the more so, the more the capital is fixed in space, e.g. as physical infrastructure. Such new investment frontiers (e.g. oil exploration and highways, or more recently ICT infrastructure) may underpin ascending political regimes; political regimes in turn shape the environment in which new technology is generated, the innovation regime.

Following this analysis, NETs may be seen as a set of technological *promises* which define and legitimise new spaces for investment—in essence, NETs can be seen as a promise of a spatio-temporal fix. Theorised as a STF (see also [5, 43, 48]), NETs can be understood not merely as a response to the climate issue, but specifically as a response of *capitalist* societies, as the scientific and political climate change problem is translated into a problem for capital and responses shaped accordingly. And so we can begin to conceptualise how capitalist interests are implicated in mitigation deterrence. Carton [5] articulated clearly how existing fossil fuel assets are physically vulnerable to the material impact of climate change, but also that their legitimacy is threatened politically, and that it is the latter crisis that is the more pressing now.

Under the original conception of STFs, it is actual investment that defers crises. However, NETs can act as a different sort of STF. At present, there are no NETs deployed at scale. Though there are funded pilots and trial schemes, actual investment is tiny, compared to the scale of NETs that is illustrated in models or projections. NETs at scale is a promise, not (yet) a reality, but it already has impacts as a promise [2] on policy and decision-making. Thus NETs actually serve as imagined future STFs and so could help defend the legitimacy of fossil capital, and defer the crisis for capital, whilst storing up more climate trouble for the future, if not followed up by large-scale successful deployment. We call this a *defensive* STF (or, less formally, a ‘technology of prevarication’). Markusson et al. [30] have argued that Carbon Capture and Storage (CCS) has long played the role of a defensive STF, and that NETs were likely to. In short, defensive STFs do not rely on material realisation but exist primarily as discursive, cultural phenomena. The mere promise of NETs is enough to defer a legitimacy crisis for fossil interests.

**Fig. 1:**
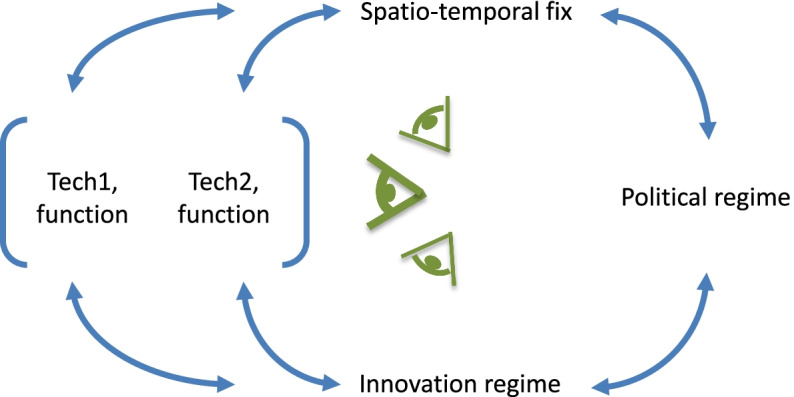
The cultural political economy of mitigation deterrence framework (adapted from [31]). Note: The eye symbols in the middle of the figure indicate the multiple possible viewpoints of the actors implicated in the system, including us as analysts. The dominating, system-conservative viewpoint, indicated in bold, strongly shapes the framing of technologies, such that they tend to fit with and support the regime. There is some scope also for alternative viewpoints to impact on the co-evolution of the system. Strategic reflexivity and material subversiveness is thus possible.

Markusson, et al. [31] combined this CPE framework with McLaren’s work on mitigation deterrence from solar radiation management, setting out a model of how mitigation deterrence risks arise from the pursuit of NETs. The paper discussed how framings of technologies shaped how substitutable they are in practice, and how the framing(s) best able to support the incumbent political regime will be favoured—although there is still scope for reflexive, strategic^4^ action and subversion (see Fig. 1). Technologies are framed as ‘substitutable’—in other words, it is claimed (or implied) that NETs can substitute for more conventional emissions reduction. This supports the dominant techno-economic framing, and mutually reinforces the policy approach of emissions trading, a cornerstone of neoliberal climate policy.

Note that defensive STFs are not specific to the neoliberal era. They are part of capitalism’s repertoire of defence mechanisms against the self-harm threatened by the ecological crises it tends to produce [14, 41], but may take different forms under different political regimes.

### The practices of the cultural political economy of mitigation deterrence

So far, we have described the CPE of mitigation deterrence as a series of relatively abstract, processes and system effects. Here we elaborate on this in terms of the more detailed practices involved in producing these effects. Carton defined the ‘political economy of delay’ to climate action as “a constellation of economic, political, cultural and everyday practices that in numerous ways serve to postpone the necessary devaluation of fixed fossil fuel capital” (2019: 765), and we here follow this cue, focussing especially on how the practices (re)produce and rely on discursive technological promises, in line with our cultural political economy of mitigation deterrence framework.

**Fig. 2:**
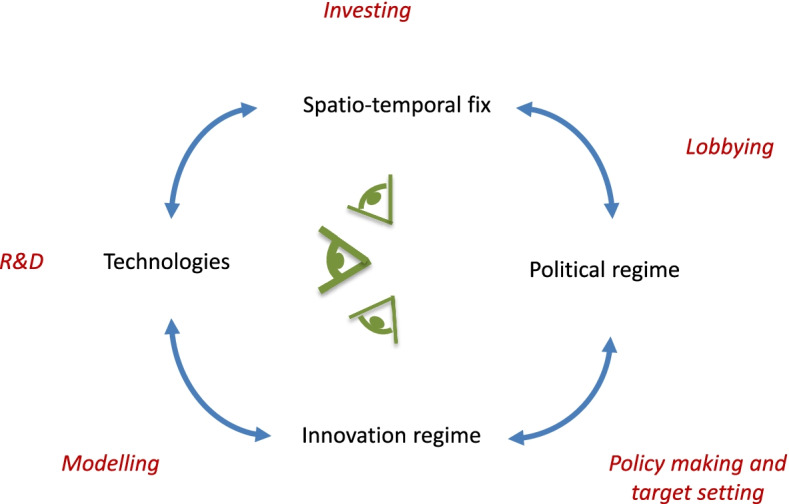
Practices of the cultural political economy of mitigation deterrence. Note: practices indicated in red italics, located approximately where they are implicated in the system.

In Fig. 2, some of the key practices [3, 44] implicated in the cultural political economy of mitigation deterrence are indicated, in red. Starting with the technologies part of the diagram, *research and development* (R&D) is a key practice producing technology promises, as well as the material experience needed for any deployment. Imagined and realised as STFs, *investment* in technology deployment is possible, and potentially profitable. The economic interests thus sustained use *lobbying*, among other means, to secure a favourable political regime, which in turn delivers the *policy making and target setting* that define the innovation regime. To close the circle, science produces not just the R&D involved in producing technology promises, but also *modelling* that helps mediate between policy making and technology development.

Whilst this is not an exhaustive account, and the circular progression of the paragraph above is a simplification of the full dynamics of a co-evolving set of practices, it is a plausible starting point for interpreting the cultural political economy of mitigation deterrence in terms of constitutive practices.

## Digging a hole: co-evolving practices of the cultural political economy of mitigation deterrence

This section further explores the structural dynamics of mitigation deterrence. Note that our perspective is a relational Marxist [25] one, where the only-ever-temporary stability of the system with its co-evolving constituent parts, and characterised by contradictions and ambiguities, needs ongoing maintenance and regularisation. We will discuss the scope for agency in the “Stopping digging: reducing the risks of mitigation deterrence” section below.

We first identify key modelling practices, and resulting framing effects that contribute to mitigation deterrence of NETs, by reinterpreting the results of McLaren [36] who engaged with modelling literature and quantified risks of mitigation deterrence from NETs. We then also discuss how the practices of the cultural political economy of mitigation deterrence co-evolve. For this, we build on McLaren and Markusson [32] who reviewed the history of low-carbon technology promises, and their co-evolution with modelling and climate policy, and showed that NETs is only one in a series of technology promises that have undermined climate action. Finally, we briefly speculate on how these practices might fare under a change away from the current neoliberal political regime.

### Practices and framing effects

Mitigation deterrence is a temporal phenomenon: the future promise of NETs replaces present-day mitigation. Not only is action deterred, and the climate crisis made more acute, but the severity of the crisis for fossil capital may even ultimately be exacerbated if the promise of NETs enables continued fossil fuel exploration and development. Mitigation deterrence thus relies on decision makers being oriented to the future in particular ways, and on systemic effects emerging over time. Here, we discuss how this happens in modelling practice.

A key route to policy impact of NETs today goes via climate pathway modelling. The function of such modelling is to suggest, or predict, future pathways to emissions reduction and climate stability, and in doing so, to inform present-day policy decisions. We can understand the potential for NETs promises to contribute to mitigation deterrence by analysing the practices that are involved in climate pathway modelling, and the temporalities at play in the process. Here we specifically discuss a tension inherent in an ambiguity between two different ways of understanding and representing NETs. NETs are, on the one hand, a possible kind of item used in the practice of *carbon budgeting*, and, on the other, a set of socio-technical *innovation practices*.

NETs came to the fore in climate policy, and the modelling that underpins it, with the adoption of carbon budgeting [35]. Artificial GHG sinks offer unique possibilities to make tight carbon budgets—and specifically a *net zero* target—palatable and plausible. This happens in two ways: firstly, NETs are seen to compensate, or net off, some residual, hard-to-remove emissions. Secondly, if the budget overshoots, i.e. if emissions reductions are not swift enough, NETs provide the promise of negative emissions in future years, to clean up. NETs therefore feature in these roles in many models. Carbon budget modelling has not just mediated between science and policy, but has had a key role in generating the imaginary [24] of NETs [1, 6].

To be useful as a carbon budget item, NETs have to be seen, at least provisionally, as a black-boxed ‘thing’ that can be planned—i.e. predicted and managed. The thinner and simpler the framing of the techniques, the more likely they are to seem predictable and manageable. Modellers make judgements about the plausibility of specific NETs, in order to include them in carbon budgets. Such judgements may contain caveats, but these are easily watered down or lost in the process of writing up results, or indeed summaries for policy makers, resulting in the “invocation of an improbable certainty about future socio-spatial relations” ([5]: 764).

An alternative to seeing NETs as a black-boxed carbon budget item (or several) is to approach them as a set of socio-technical innovation practices, embroiled in a cultural political economy. Viewed in that way, all the things that can fail, and a fuller range of impacts, are more readily visible and foregrounded [36]. Getting to widespread deployment is understood as involving a complex set of contested *innovation practice*s including research, testing, marketing, financing, hyping, protesting, lobbying etc. We here take a relatively encompassing view of innovation practices, to include not just apparently techno-economic, but also cultural and political ones [2, 15, 49]. With this rich understanding of how NETs innovation processes might unfold, it is also easier to see all the ways in which they may interact—functionally, environmentally, economically, culturally, politically etc.—emergently with their context. This includes interactions with other climate policy options, in ways that can lead to synergies or deterrence effects.

This rich view, including contestation and emergent effects, can be compared to the simplified and foreshortened step from the ‘now’ of the baseline to the temporal endpoint of a budget, as depicted in a simple graph, for example, of carbon reductions and removals over time. The modelling perspective on NETs thus leaves deterrence risks out of the frame and makes them harder to counter. Not only do the summary outputs of models conceal any substitution effects, but the specific practices of limited parameterisation of technologies (as bounded, with limited or no side effects or co-benefits), discounting of future costs and benefits, and overall financial cost-optimisation come together in ways that give integrated assessment models a strong preference for any potential future technological fix over near-term emissions cuts, and mean that rebound effects are not automatically considered. It is possible to estimate the likely scale of effects of such mitigation deterrence, in terms of emissions reductions not achieved, and consequent impacts on global temperatures if NETs are not subsequently delivered. McLaren [36] reviewed the expectations of NETs implied by integrated assessment modelling, and concluded that 371–545 Gt of carbon could be at risk from mitigation deterrence, which could add another 0.7 °C of warming.

The risk of mitigation deterrence is thus exacerbated by the way NETs are framed in carbon budgeting practice. Four framing effects^5^ can be highlighted: rationalism, certainty, substitutability and boundedness. The basic assumption of *rationally* planned and coordinated deployment, which is itself optimistic, obscures messier scenarios of ‘wild’ deployment. By including NETs in models, based on a minimum level of plausibility of their future deployability, the modelling gives the impression of a degree of *certainty* (cf. [5]). This overshadows the uncertainties of NETs innovation processes. In the context of narrowly framed climate policy goals, a limited number of modelling parameters are used to describe NETs functionality (such as GtC), thus giving an impression of NETs being readily *substitutable* for other options (cf. [7, 33]). A limited number of parameters and limiting factors are also used to describe NETs impacts and scale so as to make them modellable. This likely means an underplaying of the risk of rebounds, multiplying factors etc., giving the impression that the technologies involved are culturally and politically inert and only interact with other options through resource and cost competition, and facilitate the construction of NETs as *bounded* entities (and set of component entities). These four framing effects—rationality, certainty, substitutability and boundedness—are differentially at play in the various specific mechanisms through which mitigation deterrence from NETs occurs.

These modelling practices and the framing effects they produce are not just a matter of (eminently understandable) practical limitations to modelling; they are also implicated in the neoliberal political economy, where markets are used to govern issues, and where markets do not already exist, for the state to set them up, with emissions trading a pertinent example. There is thus a tension between plan and market in neoliberal climate policy, which adds to the ambiguities and contradictions of actually existing neoliberalism. Carbon budgeting has supported the articulation of caps and deadlines as climate policy targets, to be implemented through economic instruments like emissions trading. The credibility of the market-instrument approach relies on the planning of caps and deadlines to appear definite and certain, and so to promise to constrain the flexibility that emissions trading affords, at the same time as it is enabled and stimulated by the setting up of the emissions allowance market in the first place. In practice, for a range of reasons, including divergent national implementations reflecting domestic political economies, the flooding of the emissions markets with phony permits and financial crisis [12], capital interests have trumped planning. Framing NETs as certain, as a defensive STF promise, helps sustain the idea that decarbonisation is governable, even when other options may fail to materialise and the problem becomes more urgent. This effectively reduces the pressure on capital [5]. The modelling practices of carbon budgeting thus support neoliberal climate policy, and so the neoliberal political regime and the continued economic viability of fossil assets. The result is enhanced mitigation deterrence risks, as partial policy failure may well ensue, and NETs deployment may fail to fully materialise.

Moreover, Markusson et al. [31] argued that under a neoliberal political regime, the specific form of substitutability constructed is *fungibility*, whereby positive emissions and negative emissions from NETs are commensurated into quanta of the same kind that can be priced and traded on the same markets. Modelling practices that present NETs as bounded obscure complex externalities, and so facilitate the construction of the radically simplified characterisations necessary for commensuration.

We have here been able to detail further how specific modelling practices orient policy making to the future in particular ways, and are involved in creating and enhancing the risks of mitigation deterrence from promises of future NETs. We have seen how four framing effects (NETs as rational, certain, substitutable and bounded) contribute to sustaining the legitimacy of the neoliberal, fossil-dependent political regime and its policies. We have focussed here on modelling, but we argue below that this happens through a mutually reinforcing interaction between the practices of climate modelling (specifically carbon budgeting), climate policy-making practices (including target setting) and low carbon innovation practices (specifically NETs, and dominated by R&D, but also demonstration etc.). Note also that other practices, like lobbying, are implicated in the co-evolutionary dynamic of the cultural political economy of mitigation deterrence too, but not discussed here.

### The evolution of the cultural political economy of mitigation deterrence

An analysis of the history of climate science and policy shows that mitigation deterrence has had many previous manifestations, and is not just an issue for NETs. McLaren and Markusson [32] mapped the history of climate policy in five phases, and related this to developments in modelling and the prominence of a range of technology promises, drawing mainly on international and UK material. Each phase has two distinct characteristics: a change in conceptualisation of the overarching goal of avoiding dangerous climate change, or ‘target framing’; and shifting promises of future technology deployment. Working together, these have enabled the avoidance of transformative social, economic and political change, and therefore delayed decarbonisation. Target framings have co-evolved with modelling practices, in part reflecting growing computational power, and science and engineering research and development frontiers, resulting in an expanding set of technology promises. Rather than lead to technology development and deployment, however, the outcome has primarily been a deferral of action on decarbonisation.

Target framings have evolved [35] from a target of general ‘stabilisation’, via ‘percentage emissions reductions’, ‘atmospheric concentrations’ and ‘carbon budgets’, to ‘outcome temperature’; a sequence that, after the initial vague stabilisation frame, has moved from cause towards impact on climate (understood as globally averaged conditions). With the transition from emissions reductions to atmospheric concentrations in the late 2000s, enabled by improved modelling, came interest in a technology that can act on GHGs already in the atmosphere. Here, BECCS technology was forged and given a role in scenarios, drawing on earlier CCS and biomass modelling and evidence. Somewhat later, carbon budgets became the favoured target framing, drawing again on modelling improvements, as well as (changing) technology promises. With carbon budgeting, the notion of negative emissions came into its own, offering both ways of balancing out recalcitrant emissions and reversing overshoots. More recently, climate policy targets have been set in (global average) temperature terms, opening up potential demand for technologies that act “directly” on “temperature”. In this regard, we would expect Solar Radiation Management (SRM) to be the next promise lined up to make the policy goals achievable without radical economic disruption—with NETs remaining necessary too—though suffering from disappointment after the initial hype. Similar stories can be told about other, earlier climate response options, like nuclear energy and fossil CCS.

All of these technologies have predominantly been constituted as defensive STF promises. Despite (fluctuating) R&D investments, deployment has in practice mainly been partial and geographically patchy, and in the case of carbon sinks maybe even gone backwards with net carbon releases from land. Under the neoliberal regime, climate policy has favoured words and trading over action. Surprise [48] writes about SRM as a pressure valve that makes the pressure on fossil capital manageable. As our historical overview shows, there is an organ-ful of such valves already mounted and in play. Though one or a few may have dominated policy discussions at a given time, previous ones have not disappeared, but become taken for granted in policy and modelling. Each technology promise has gone through a hype-disappointment cycle leaving behind mainly modelling traces (cf. [40]: 254). The climate models contain sedimented layers of old technofix promises. Together they serve as a historical record and measure of the unmanageability of the climate crisis under conditions in which responses remain constrained by a strong priority to avoid disruption and preserve economic, political and social business as usual.

In terms of understanding the cultural political economy of mitigation deterrence, we note that the set of practices (policy making and target setting, modelling and R&D), involved in enabling deterrence and delay from NETs, are also evident in other examples of climate-related technological promises. Moreover, the practices of the set have co-evolved, and technology promises—as defensive STF promises (or less formally, ‘technologies of prevarication’)—have evolved with them. As targets shifted from cause (emissions) to effect (temperature), technology promises became further removed from the sectors where emissions originate, such as energy, transport and land use; and so they become further removed from challenging the value of existing carbon-dependent assets, and so the underlying capitalist logic of society. This, more or less deliberate, avoidance of challenging carbon-dependent capital interests goes a long way towards explaining the intense, felt attraction of each generation of technology promises, and the deterrence they enable.

The set of practices has thus evolved in a specific direction, which has allowed the decarbonisation of the economy to be deferred (or done very slowly and partially), relying on imagined technologies that are ever more invasive in the natural environment (and ever less so in the economy), benefitting primarily global elites and corporate interests. Under their dominance, a hole has been dug for us all to live in, using defensive STF promises as shovels.

And there is not obviously a technological endpoint to this progression. Even if powerful actors decided to, prepared to, and perhaps even tried to ‘regularise’ the global temperature directly with SRM, there could be reasons to prepare to fix the impacts of SRM deployment with further new technology, and so on. It is impossible to foresee exactly what STF might be offered to supersede SRM, but the sequence so far would suggest for example a (perhaps nano-) technology imagined able to compensate for and remediate climate change without any of the side-effects of currently envisaged SRM.

However, there are reasons for cautious hope that do not simply rely on such imagined technical fixes. Although, with the rise of temperature framed targets, SRM looks like the next likely promise to prop up climate policy, and the political regime with it, the future may be less predictable than that. Currently, the neoliberal regime is undergoing yet another moment of crisis. The financial crisis of 2009 shook the regime, but it has sustained itself through initiatives such as quantitative easing. Even as stock markets have continued to grow, COVID-19 related impacts on economic activity and relations have added to the stresses facing neoliberalism. What has so far remained largely talk, might under a different political regime turn real. There may be a resurgence in low carbon innovation, maybe induced through Green New Deal-like initiatives under a more progressive regime. Or, the current trend towards nationalist protectionism may mean governments, for example the Chinese one, seeing merit in investing in technological protection of their own nation from climate disasters—though there may also be resurgent SRM under protectionist regimes [32].

**Fig. 3:**
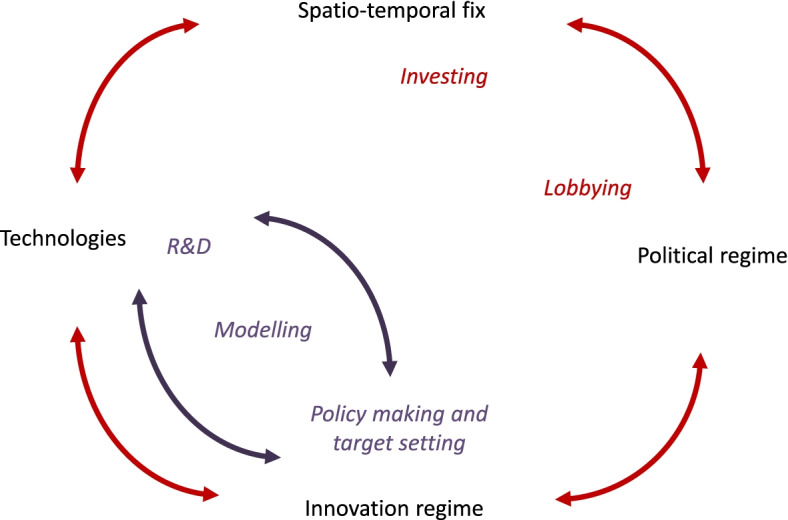
Fast and slow co-evolutionary dynamics of the cultural political economy of mitigation deterrence. Note: The quick, small, co-evolutionary dynamic is indicated in purple, and the slow, large loop in red. Related practices indicated in italics

There may be mitigation deterrence dynamics playing out through defensive STFs under different political regimes, though they would operate differently and perhaps with different intensities [37]. NETs techniques are no silver bullets, but there are no simple political solutions either. However, the financialisation of the economy under neoliberalism is likely to be followed by a round of investment in the “real” economy, assuming that capitalism as such survives and that the long waves of system renewal do not stop rolling [28]. The defensive STF promises proliferating under neoliberalism in response to climate change may well see their fortunes change, and receive substantial amounts of investment, and so turn into classical STFs under a new capitalist phase. The (comparatively fast) co-evolutionary dynamic of mitigation deterrence would then turn into part of a larger (and slower) co-evolutionary loop of regime change (see Fig. 3).

Summing up, the process and practices enabling mitigation deterrence from NETs are generalisable as a cultural political economy of mitigation deterrence. The practices have co-evolved, in a direction that has allowed ongoing prevarication, re-focussing technology development efforts from emissions towards the atmosphere, and the digging of an ever-deeper climate crisis hole. This discussion helps inform our analysis in the next section of what can be done to *stop* the digging and begin to do something more constructive. This includes examination, in line with our phronetic take on practices, of the scope for reflection on how the practices involved are performed and the emotional labour that entails.

## Stopping digging: reducing the risks of mitigation deterrence

This section is about what agency there is to deal with mitigation deterrence in practice. We draw mainly on results from a series of deliberative workshops, which were designed to facilitate strategic reflection on mitigation deterrence from NETs, and how it might be countered [37]. First, we introduce the themes of phronesis and emotion, and their relation to the cultural political economy of mitigation deterrence, in abstract terms. We then discuss the emotional labour necessary for reflecting on and changing one’s involvement in practices that contribute to mitigation deterrence from NETs whilst continuing to believe that NETs will be needed alongside other climate crisis responses. Finally, we specify practice changes that could help reduce the risks of mitigation deterrence, and the scope for such change to be effective during and after the neoliberal political regime.

### Phronesis and emotions

From our perspective, all actors, including us as analysts, are implicated in the cultural political economy of mitigation deterrence (cf. Fig. 1 above). Actors are here understood as internalising the legitimacy of the hegemonic political regime, and can be expected to be committed to its preservation [29] and that of capitalism more generally [18]. Under neoliberalism, specifically, that hegemony has been built around a widespread belief in markets as the best tools for solving problems, and innovation as best governed by markets (in spite of rhetoric about market failure). Under capitalism generally, ideas for new technologies are almost always imagined as eventual commodities, and tend to be configured as incremental commercial opportunities by the operation of venture capital [17]. As a result, they tend to operate as spatio-temporal fixes (as we discussed in the “Cultural political economy and the lure of spatio-temporal fixes” section). The neoliberal hegemony is not absolute, and our CPE perspective does not preclude contestation. In particular, this hegemony is strong in practice in climate policy, and alternative solutions are poorly represented in modelling [21]. But in the wider societal debate there are challenges to the neoliberal common sense, for example the Green New Deal or degrowth. Altogether, then, our subjectivities, and so our agency, are thus implicated in a system prone to mitigation deterrence. A diverse range of actors, clearly including NETs technical experts, climate modellers and climate policy makers, but also a wider set of stakeholders such as fossil fuel industry, finance, media and lay people, are involved in the circulation of climate narratives and technology promises. As NETs researchers, we are implicated too—we are all observing the system from *within* it.

Our approach to analysing agency draws on the notion of phronesis [13, 27, 46]. With Tyfield [51], we define phronesis as “a form of wisdom-cultivating knowledge practice, regarding … living together in complex, dynamic socio-techno-natural systems”. In line with this, we understand all actors in the system as strategic, emotional beings, operating dynamically in interaction with others, and in settings structured in turn by power relations. Knowing the system, and knowing what to do about its problems, involves not just disembodied, dispassionate techno-economic knowledge-making, but also strategic attention to political and normative issues, as well as emotional labour.

All the practices of the cultural political economy of mitigation deterrence (R&D, modelling, policy making etc., cf. Fig. 2) involve phronesis. Climate professionals and lay people alike [20, 39] have to, more or less wisely and effectively, judge what is feasible and doable for themselves, and manage their emotions to retain hope and avoid despair. When faced with a recalcitrant, high-fossil political economy, actors may judge only technical fixes feasible, and need to ignore associated risks (and alternative solutions) to be able to stay optimistic. Head and Harada’s [20] study of climate scientists found that scientists tended to focus on ‘best case’ scenarios because they found it difficult to be confronted with the realities of climate impacts. Similarly, Willis [53] noted that politicians tend to play down the significance of climate, in an attempt to fit it within their existing political outlook. Below, we discuss the felt attraction of delay that technology promises have offered both modellers and policy makers especially, in the current political economy.

It should not be surprising that feelings matter. Recent discussions about ‘eco-anxiety’, following high-profile and impactful campaigning by school children and Extinction Rebellion, as climate change became a more prominent topic for many, testify to the emotional impact of taking climate change seriously [9]. Under a neoliberal social and economic order, many, and especially white people in the Global North, tend to be socialised into placing their hopes in techno-fixes, especially those funded through market instruments. Starting to question those hopes is difficult and can be painful. (Whereas others, including some indigenous groups, have had to face up to anthropogenic climate crisis for a long time already—see [52]).

Professional actors also face pressures not to be overt about their strategic judgements and emotional tactics, which makes it harder to have open discussions about the risks of dominating climate options, and alternative solutions. Below, we discuss our use of phronetic, deliberative methods as emotional practices [45] that elicit and prompt reflection on the strategic positioning and emotional labour involved in facing up to the risks of mitigation deterrence from NETs promises.

### Painful reflections on life in the hole

We engaged with stakeholders through a set of deliberative workshops, designed to explore scenarios unfolding to 2050 of mitigation deterrence under varying alternative assumptions about the evolving political future [37]. Some participants were NETs experts, but not all. Most were professionally engaged in addressing the climate crisis. A few participants were master’s students, with an interest in climate, but less strongly formed commitments to particular ways of seeing and working with it.

We undertook this research with a commitment to be directly ‘useful’, to produce advice for action on how to reduce mitigation deterrence risks through changed practices, rather than just be ‘merely’ critical. The workshops were designed to promote explicit reflections of both a professional and a more private nature, and to reflect collectively on what would count as good outcomes, adopting a phronetic approach [13] to deliberation. In exploring the scenarios, the NETs stakeholders were prompted to reflect on how they go about performing the various practices involved in (what was simultaneously being clarified as) the cultural political economy of mitigation deterrence, and thus about what it is like to live in and help dig the hole. We hoped to access not just narrowly ‘techno-economic’ understandings of what counts as (collectively) rational behaviour in the face of climate change, but also normative, emotional and political dimensions, to understand what values, motivations and interests are at play; perhaps starting with a recognition of ways in which everyone is (at least somewhat) implicated and complicit in ‘digging the hole’ and so deciding to stop. We also encouraged the group to use these insights to develop recommendations for how to minimise mitigation deterrence risks. Many, though not all, workshop participants agreed about the plausible existence or severity of mitigation deterrence risks from NETs. Agreement typically grew during the workshops, in part because we introduced (as we had thought) plausible mitigation deterrence mechanisms gradually, and often after vigorous discussion.

The ways in which some participants (not just NETs specialists), mainly initially, rejected the prospect of mitigation deterrence from NETs reveal interesting things about ‘life in the hole’. Rejections were often based on apparently objective arguments, about, for example, expectations regarding how various actors would behave, or how relevant markets would work. But there were also overtly *emotional* aspects to the rejections. A plausible interpretation is that the stakeholders, who were typically well-versed about the severity of the climate crisis and the difficulty of responding adequately to it, had invested hope in the promise of NETs and found the thought of mitigation deterrence risks arising from it painful to contemplate (cf. [20, 39]), since such thoughts may seem to threaten belief in precious (i.e. much-needed, scarce and highly-valued) ‘ways forward’. Such ambivalence was sometimes expressed as initial rejection of the idea of mitigation deterrence, but could turn into acceptance, especially with reassurance that the purpose of the discussion was not to criticise NETs, but to identify the cultural, political and economic conditions under which they could be most useful. This process of opening up the problem thus entailed emotional labour, as ambivalent feelings about NETs, and about the political economy, had to be processed. Acknowledging mitigation deterrence risks is not just a matter of cognitively realising that some aspects of NETs and society are problematic, but an emotionally heavily loaded matter of surrendering commitments to particular versions of NETs and society. Insofar as one’s hopes and plans depend on the continuation of the current society, letting go of it, and NETs imaginaries design to fit it, is personal and painful (cf. [22]).

We observed that many participants felt a need first to express their own commitment to NETs, before being able to countenance the possibility of mitigation deterrence from NETs. This ‘obligatory passage point’ [4] phenomenon can be interpreted as a way of dealing with the ambivalence of both placing hope in NETs and fearing its potential deterrence effects. But, as noted above, talking about the risk of mitigation deterrence can be seen as an attack on NETs, and first expressing support for it can also be interpreted as an act of social boundary drawing. It can be read as an implicit act of affirming a position and identity of being among those who support the NETs project (seen as an essential component of managing climate change), as opposed to those who do not. And insofar as NETs is seen as risky but necessary, being a nay-sayer is unreasonable and irresponsible. Moreover, when NETs is understood as necessary to defend society, supporting the technology is an act of affirmation of loyalty to that society, at least temporarily, seeing tackling climate change as a more urgent task than transforming the political economy. That society was typically understood in general terms as liberal Western market democracies and our ways of life under it, and participants generally recognised its capitalist foundations, if not explicitly its current neoliberal form (cf. [29]). Seeing NETs as necessary to uphold our current society reflects awareness of how NETs works as a defensive STF. There was definitely awareness about how NETs would work as a defensive STF once deployed, and during the workshops the awareness also grew of how it might operate to deter mitigation. This social boundary drawing also sets a limit to how much NETs proponents can afford to worry about mitigation deterrence risks. Those so worried about deterrence that it outweighs their support for the technology can then be suspected of naivety and excessive radicalism.

We had set up the workshops to be forums for recognised stakeholders in a relatively broad sense including civil society organisations, but which stopped short of direct action protestors or militant activists. The workshops more naturally engaged with ideas of reform than revolution. So in this sense, the obligatory passage point was partly a result of our pragmatic research design, and another project may usefully explore more politically radical ideas to counter deterrence. However, we can also interpret this observation as evidence of a very real concern for the future of our capitalist society among establishment stakeholders, and insofar that action on mitigation deterrence is dependent on their support, it may come best from ‘critical friends’ of NETs and (neoliberal) capitalism.

The workshop process was not, however, a matter of rigidly policing and reproducing a boundary between what is acceptable and thinkable and what is not. Once past the obligatory passage point, ambivalence about NETs could be admitted, and a fuller problem description including strategic reflection on how the political economy is implicated in mitigation deterrence could be discussed. And with this, new possibilities became thinkable too. Better ways of doing NETs, with less risk of mitigation deterrence, requiring changed policies that clash with currently taken for granted market-oriented principles, were discussed. Some participants argued that this would be easier to do with a different political economy, whereas others (even if reluctantly) focused on urgently mobilising feasible tools within the current political economy.

### What can we do?

The workshops helped us formulate recommendations for how policy could be developed to reduce mitigation deterrence risks. These recommendations can be summarised under four headings: separation, accountability, wide focus risk assessments and phronetic deliberation. Each counters one of the problematic framing effects identified above—substitutability, certainty, boundedness, and rationalism, respectively. We outline the first three here (the fourth is addressed later in this section):

1. Separation as a policy principle would require, among other things, setting separate policy targets for NETs [34]. This would reduce the risk of NETs being *substituted* for mitigation.
2. Accountability, by having effective systems in place to avoid double counting and cheating, is about acknowledging some causes of the deep uncertainty of innovation processes. Together with policy support for innovation, this would help reduce the risk of *failure*, and improve the *certainty* of NETs outcomes.
3. Doing multi-criteria assessments [16] that take a wide range of effects into account, including mitigation deterrence, is about acknowledging the externalities that are made invisible by assumptions of tight *boundedness*.

These recommendations may seem eminently sensible and straightforward. However, one reason that they would be challenging to implement is that they would highlight and reduce the functionality of NETs as mitigation deterrence, thereby exposing the financial and other interests who are benefiting from mitigation deterrence. This brings us back to the question of: *under what (political economic) conditions can NETs coexist with, and complement, other mitigation strategies?* Can it realistically happen under neoliberalism? What would the world have to look like for our recommendations to be taken up and acted on? What dominant political regime would it have? In Markusson et al. [30], some of us contrasted the unlimited faith in markets during the neoliberal phase of capitalism [38] with earlier classically liberal and social liberal phases where limits to capital were more clearly recognised [50], legitimising public sector planning and/or non-market interventions including regulation and nationalisation. Moreover, in the post-War socio-liberal phase the balance between labour and capital had shifted somewhat, so that social reproduction (health care, education, rest and recuperation etc.) and to some extent environmental reproduction (at least for some locations and scales^6^) were more strongly defended against market domination than previously (and subsequently).

All of the framing effects contributing to mitigation deterrence identified in this paper are tendencies under capitalism generally, but there are also some differences in emphasis and form, under different phases. In particular, given its characteristic faith in markets as optimal decision-makers, under neoliberalism each of the framing effects assumes a particular form and is likely also strengthened:

- *Substitution*—with the emphasis firmly on the success of technologies in markets for narrowly-specified and quantitative functionality (e.g. ‘total GHG emissions/savings’), neoliberalism privileges consideration that compares (socio-)technological options as highly substitutable, while in earlier phases of capitalism fewer things were commodified and exposed to markets.
- *Certainty*—likewise, trusting in the delivery of collective goals through competitive success of privately provided (new) technologies, neoliberalism cultivates a particular non-chalance or confidence regarding the capacity of entrepreneurial innovation to deliver ‘what people want’, and hence a presumed certainty that technological promises can and will be delivered, even as planning for those outcomes is seen as impossible or ineffective and as some collective problems persist.
- *Bounded*—finally, the two characteristics of neoliberalism just mentioned (i.e. faith in markets to deliver technologies that then are optimal interventions in social problems, and the flipside of confident agnosticism about the future) combine in the form of deliberate lack of interest in the follow-on effects of new technologies as they are adopted *en masse*—because it is for the market, not human intelligence (individual or collective), to decide best how things work out, and on an ongoing and never-ending basis. New technologies, therefore, may—indeed, *should*—be treated as bounded, since to conceptualise them more searchingly is to overstep the legitimate bounds of human cognition, trespassing on decisions that are much better left to the market.

At the very least, then, we can say that the drivers behind mitigation deterrence are likely strong under neoliberalism, and that other regimes of capitalism that manifest weaker or looser conditioning of all or any of these frames may be preferable in this respect. For instance, both social and classical liberal regimes preserve much bigger roles in optimal decision-making for accountable evidence and argument, which may in turn make them more amenable to becoming alert to the unintended negative (side-) effects of decisions made using these frames, as set out here.

The fourth and final policy principle about deliberation involves acknowledging the normative, emotional and political dimensions of living well with the attraction of delay, and so corresponds to the fourth problematic framing effect of *rationalism*. Our phronetic approach helps us deconstruct the NETs STF, and maybe (we hope) re-construct it in ways that make it less prone to cause deterrence and delay. The approach opens up the topic of NETs so that mitigation deterrence risks become visible, thereby providing scope for efforts to deliberate and educate, and so some hope of changing enough hearts and minds for reform to the prevailing NETs strategies and policies, i.e. those currently at risk of causing mitigation deterrence.

These are encouraging signs that a phronetic approach to deliberation on mitigation deterrence can produce insights about the problem, suggestions for what to do about it, and some transformation of hearts and minds well placed to make a difference. Our workshops worked well as experimental emotional practices [45]. We take this as support for our call for further phronetic deliberation, whilst also noting that getting the discussion to move beyond the dominant, rationalist, economistic social imaginary, and achieve phronetic deliberation, required us to have a relatively complicated workshop design, and asked quite a lot of participants. Limitations of the workshop design are discussed in more depth in a separate paper [37].

## Conclusion

Above, we re-interpreted the cultural political economy of mitigation deterrence in terms of practices, and refined the notion of defensive spatio-temporal fixes. This enabled us to identify key practices contributing to mitigation deterrence, through a set of framing effects, and discuss how these practices have evolved as part of the neoliberal cultural political economy, producing a sequence of poorly adopted technological promises for tackling climate change that primarily functioned as defensive spatio-temporal fixes. This evolving dynamic has caused a perverse depoliticisation of future expectations, whereby effective responses to climate change embodied in promises of carbon removal, framed in particular ways, empower continued procrastination and prevarication in the present, as the technologies are, often unreflectively, enrolled into the preservation of neoliberal capitalism.

We further discussed the scope for changing the practices and the system they are part of to reduce the deterrence risks. We argued that both understanding and reducing those risks require phronetic knowledge practices, involving not just disembodied, dispassionate techno-economic knowledge making, but also strategic attention to political and normative issues, as well as emotional labour. Reflecting on the hole we have dug for ourselves is painful.

The paper synthesises and builds on previous work on mitigation deterrence, particularly in analysing how modelling practices contribute to the risks of mitigation deterrence through framing effects; and in identifying the emotional dimensions of reflecting on those risks, which in turn influence practices. We have also provided concrete policy recommendations, but more fundamentally we hope our findings help refocus climate policy and research away from yet more new promises of future technological solutions, instead seeking to reveal and to understand better the cultural, politico-economical and affective reasons why potentially effective responses are not adopted. That way, we can maybe climb out of the hole.

Our work was done by relatively affluent academics in the Global North, based on engagement with NETs stakeholders with similar characteristics, with the privilege of being able to take the time to discuss, think and feel about climate change under comfortable conditions. There would be room to develop the phronetic approach further, by engaging with those less favoured by neoliberalism, and less guilty of digging the hole, than affluent NETs stakeholders and academics, and, perhaps relatedly, for exploring how more radical (in the sense of unruly, disruptive, revolutionary) political stances might help inform the analysis of NETs as STFs, and the resistance to decarbonisation delay. Additionally, we have paid particular attention to the practices of climate modelling, and there is scope for more detailed studies of how other practices, including notably investments and lobbying, contribute to mitigation deterrence.

## Data Availability

In line with the consent provided by workshop and interview participants, to protect personal identities, raw data cannot be made available. An anonymised subset of the transcribed material has been provided as supplementary information to McLaren et al. [37]. The data for the quantification of how much carbon is at risk of mitigation deterrence is available as supplementary material to McLaren [36].

## Abbreviations

BECCS: Bioenergy with carbon capture and storage
CCS: Carbon dioxide capture and storage
CPE: Cultural political economy
GGR: Greenhouse gas removal
GHG: Green house gas
Gt: Gigatonne
GtC: Gigatonne of carbon
ICT: Information and communication technology
NETs: Negative emissions techniques
R&D: Research & development
SRM: Solar radiation management
STF: Spatio-temporal fix

## Footnotes

1 NETs is broadly synonymous with Greenhouse Gas Removal, GGR. Carbon Dioxide Removal, CDR, is similar but focussed on CO_2_.

2 Political regimes are here understood as assemblages of ideologies, interests and political practices, with some temporal durability, underpinned by economic blocs, and centrally placed in power relations such that they shape subjectivities, institutions and other practices elsewhere in the system.

3 STFs thus have spatial effects (cf. for example [43] for the case of NETs), and that is certainly the case for NETs (see for example [11]), but we are in this paper primarily concerned with temporal aspects of NETs as STFs, cf. Tyfield [49], because mitigation deterrence is about risk of current emissions reduction being deterred, with reference to future technology deployment.

4 ‘Strategic’ in here used the sense of having regard to wider longer-term or overall aims and objectives and the means to achieve them, as opposed to a more myopic focus on immediate or limited aims. Note that these aims need not be self-centred, but can be about the common good (as seen by the actor).

5 Identified through re-analysis of the types of mitigation deterrence outcomes in McLaren [36].

6 Even as, at global scale, this is also the moment of the ‘Great Acceleration’ [47].
